# In vitro cultivation of primary intestinal cells from *Eisenia fetida* as basis for ecotoxicological studies

**DOI:** 10.1007/s10646-021-02495-2

**Published:** 2021-11-17

**Authors:** Simon A. B. Riedl, Matthias Völkl, Anja Holzinger, Julia Jasinski, Valérie Jérôme, Thomas Scheibel, Heike Feldhaar, Ruth Freitag

**Affiliations:** 1 Process Biotechnology, University of Bayreuth, 95440 Bayreuth, Germany; 2 Animal Ecology I, Bayreuth Center of Ecology and Environmental Research (BayCEER), University of Bayreuth, 95440 Bayreuth, Germany; 3 Biomaterials, University of Bayreuth, 95440 Bayreuth, Germany

**Keywords:** Earthworm, *Eisenia fetida*, Environmental pollutants, Microplastic, Cytotoxicity, Primary cells

## Abstract

The earthworm *Eisenia fetida* is a commonly used model organism for unspecific soil feeders in ecotoxicological studies. Its intestinal cells are the first to encounter possible pollutants co-ingested by the earthworm, which makes them prime candidates for studies of toxic effects of environmental pollutants on the cellular as compared to the organismic level. In this context, the aim of this study was to demonstrate the suitability of preparations of primary intestinal *E. fetida* cells for in vitro ecotoxicological studies. For this purpose, a suitable isolation and cultivation protocol was established. Cells were isolated directly from the intestine, maintaining >85% viability during subsequent cultivations (up to 144 h). Exposure to established pollutants and soil elutriates comprising silver nanoparticles and metal ions (Cu^2+^, Cd^2+^) induced a significant decrease in the metabolic activity of the cells. In case of microplastic particles (MP particles), namely 0.2, 0.5, 2.0, and 3.0 µm diameter polystyrene (PS) beads as well as 0.5 and 2.0 µm diameter polylactic acid (PLA) beads, no active uptake was observed. Slight positive as well as negative dose and size dependent effects on the metabolism were seen, which to some extent might correlate with effects on the organismic level.

## Introduction

The earthworm *Eisenia fetida* is a commonly used terrestrial model organism in ecotoxicological research. As soil feeders, earthworms like *E. fetida* unselectively ingest soil and therefore also any environmental pollutant included therein. Consequently, their intestinal tissue is directly exposed to these foreign materials, and it is commonly assumed that effects of common pollutants such as metal ions (Nahmani et al. 2007; Sivakumar 2015) or nanomaterials (Garcia-Velasco et al. 2016; Kwak et al. 2014) on the organismic level such as mortality, reduced growth rate and reproduction, are mediated by damage to the gut cells and tissues. However, organismic reactions are complex, and understanding effects mechanistically can be challenging. An important aspect of deconvolving the overall effects is the identification of the response on the cellular level using cell lines or primary cells (Revel et al. 2021). Primary cells isolated from a specific tissue initially possess characteristics comparable to those of the cells in vivo and reflect their physiological state and reactions. This enables studies of effects on the cellular level, which are more representative for the in vivo situation than experiments with established cell lines. Moreover, such studies are also possible in case of organisms and tissues, for which no established cell lines exist.

In case of *E. fetida*, no cell lines or standard procedures for the isolation and cultivation of primary intestinal cells are currently available. Protocols do exist for the isolation and cultivation of various earthworm coelomocytes, i.e., the phagocytic leukocytes in the coelom which are the established primary cell type for ecotoxicological or immune response studies (Diogène et al. 1997; Eyambe et al. 1991; Fuller-Espie et al. 2015; Garcia-Velasco et al. 2019; Stein and Cooper 1981; Toupin et al. 1977). The respective culture media are mostly based on L-15 medium with various supplements and adjusted osmolalities (Bilej et al. 1990; Irizar et al. 2014; Roch et al. 1975; Toupin et al. 1977). However, these macrophage-like cells are physiologically and functionally different from intestinal cells and the direct compatibility of protocols for their isolation and cultivation with the needs of the intestinal cells is unlikely. In case of primary intestinal cells from the earthworm *Pheretima aspergillum*, Schneider´s Drosophila Medium (SDM) was shown to support proliferation (Gong et al. 2014), while Hansen S-301, a formulation based on SDM, has previously been used to keep tissue fragments of *E. fetida* in culture (Battaglia and Davoli 1997).

In recent years, pollution of the environment with microplastic (MP) became a matter of global concern. MP is defined as any plastic piece between 1 and 5000 µm in size. Soils in particular have been reported to represent MP sinks (Büks and Kaupenjohann 2020; He et al. 2020; Piehl et al. 2018). MP can enter terrestrial habitats via various pathways, including natural precipitation (rain, snow), illegal waste deposition, sewage sludge and wastewater, agricultural practices (plastic foil for mulching), or in some cases even organic fertilizer (Chae and An 2018; Weithmann et al. 2018). Several studies have already reported direct or indirect negative effects of MP on earthworms. For *Eisenia andrei*, histopathological evidence for gut tissue damage and responses of the immune system after exposure to polyethylene MP particles was shown (Rodriguez-Seijo et al. 2017). In *E. fetida*, MP exposure led to an increase in the organisms’ oxidative stress levels (Chen et al. 2020; Rodríguez-Seijo et al. 2018; Wang et al. 2019). However, aside from the obvious tissue damage, the putative influence of MP particles on the intestinal cells has neither been demonstrated nor excluded. This knowledge would be important for a better mechanistic understanding of how MP affects the cellular as well as the organismic level.

The aim of the study was to prove the suitability of using intestinal primary cells of *E. fetida* earthworms for ecotoxicology studies using Ag nanoparticles and metal ions (Cu^2+^, Cd^2+^) examples. Our protocol effectively yielded primary cells of high viability during short-time cultivation. Cytotoxic effects were determined using an adapted assay for metabolic activity. Subsequently, the effect of polystyrene (PS), as representative of commodity polymers, as well as polylactic acid (PLA), as an example for biodegradable polymers, MP particles on the metabolic activity of the cells was determined as well as their putative cellular uptake.

## Materials and methods

### Materials

Cell culture materials were obtained from Greiner Bio-One International GmbH (Frickenhausen, Germany). If not otherwise indicated, cell culture solutions and supplements (L-glutamine, HEPES ((4-(2-hydroxyethyl)-1-piperazineethanesulfonic acid), penicillin, streptomycin, amphotericin B) and DPBS (Dulbecco´s Phosphate Buffered Saline) were obtained from Biochrom AG (Berlin, Germany). L-15 medium was purchased from Lonza Group AG (Visp, Switzerland) and Schneider´s Drosophila Medium (SDM) from Fisher Scientific GmbH (Schwerte, Germany). Sigma Aldrich (Taufkirchen, Germany) was used the supplier for chemicals such as galactose, lactalbumin hydrolysate, tetracycline, cell culture grade water (for medium preparation) and FCS (Fetal Calf Serum). Gentamycin was obtained from Biowest (Nuaillé, France). Ultrapure water for buffer preparation was produced by a Millipore unit (Synergy Water Purification System, Merck KGaA, Darmstadt, Germany). Collagenase type II from *Clostridium histolyticum* (CLS II, #C2-28, Lot Number 47N17872A, 280 U/mg) was purchased from Biochrom AG. MTT (3-(4,5-dimethylthiazol-2-yl)-2,5-diphenyltetrazolium bromide) reagent was received from Alfa Aesar (Ward Hill, Massachusetts, USA).

Sterile filters (0.2 µm cellulose acetate) were purchased from VWR International (Darmstadt, Germany) and cell strainers from pluriSelect Life Science (Leipzig, Germany).

### Silver nanoparticles, metal ions, and microplastic particles

Silver nanoparticles with a size of 40 nm were obtained from Alfa Aesar (Ward Hill, Massachusetts, USA) and supplied at a concentration of 20 µg/mL and an absorption maximum at 416 nm in 2 mM sodium citrate buffer (#J67090). The Eppendorf Concentrator 5301 (Eppendorf AG, Hamburg, Germany) was used to prepare a more concentrated stock solution at 45 °C for 60 min (65 µg/mL in 6.5 mM sodium citrate) for use in the viability assays, see below. CuCl_2_ and CdCl_2_ were dissolved at a concentration of 100 mg/mL in cell culture grade water and the solution was sterilized by filtration (0.2 µm cellulose acetate). Polystyrene (PS) MP particles were obtained from Polysciences Inc. (Warrington, Pennsylvania, USA) with fluorescence (yellow green, excitation 441 nm, emission 486 nm) and without and with average sizes of 0.2 µm (#07304-15 and #17151-10), 0.5 µm (#07307-15 and #17152-10), 2.0 µm (#19814-15 and #18338-5), and 3.0 µm (#17134-15 and #17155-2). Polylactic acid (PLA) MP particles were obtained from Micromod Partikeltechnologie GmbH (Rostock, Germany), with fluorescence (green, excitation 502 nm, emission 527 nm) and without and with average sizes of 0.5 µm (#11-00-502 and #51-00-502) and 2.0 µm (#11-00-203 and #51-00-203). All MP particles were delivered as aqueous suspension at a concentration of 2.5% (w/v) for the PS particles and of 1.0% (w/v) for the PLA particles. The particles were declared as “plain” without surface modification, but, according to the manufacturer, they had a slightly negative surface charge due to residual sulphate ester groups.

### Buffers

M-HBSS (Modified Hanks Balanced Salt Solution) without GGE (Guaiacol Glyceryl Ether) (pH 7.25, 210 mOsmol/kg) was prepared in house according to a previously published protocol (Diogène et al. 1997) replacing GGE by NaCl to assure a constant osmolality. When indicated, GGE was supplemented to the buffer at a final concentration of 50.4 mM using a concentrated stock solution (500 mM). LBSS (Lumbricus Balanced Salt Solution) (pH 7.3, 171 mOsmol/kg) was prepared in house as previously published (Stein and Cooper 1981). Both buffers were sterilized by filtration (0.2 µm cellulose acetate). The detailed composition of both buffers is given in Table S1.

### Handling and rearing of *Eisenia fetida*

*E. fetida* were kept as synchronized laboratorial cultures under controlled conditions (temperature constant 15 °C, 70% moisture, photoperiod 16 h light, 8 h darkness) in worm composters filled with dampened soil mixed with sphagnum peat. Every week the cultures were fed with oatmeal and wormfood (Superwurm e.K., Düren, Germany).

### Production of cell-free worm filtrate (WF)

A cell-free worm filtrate (WF) of *E. fetida* for media supplementation was produced in house as follows. About 30 mature worms were washed to remove external soil and transferred into a sterile culture dish covered with moistened filter paper to naturally void their intestine. After 24 h, the worms were pooled and the wet weight was determined. For anaesthesia the worms were incubated at −20 °C for 10 min. 50 mL M-HBSS was added to 10 g of earthworms and the mix was homogenized in a hand blender. The homogenate was pressed through a 70 µm cell strainer with the help of a syringe piston collecting the flow through on ice. To remove any remaining tissue and solids, the filtrate was centrifuged (3990 × *g*, 2.5 h, 4 °C) and the supernatant aliquoted in 2 mL reaction tubes to be stored at −20 °C.

### Preparation and isolation of the intestinal tracts from *Eisenia fetida*

Mature earthworms intended for the isolation of primary intestinal cells were transferred into a sterile culture dish covered with moistened filter paper 24 h before the procedure to naturally void their intestine. To prevent bacterial and fungal growth, M-HBSS was supplemented with 100 U/mL penicillin, 100 µg/mL streptomycin, 60 µg/mL tetracycline, 50 µg/mL gentamycin and 2.5 µg/mL amphotericin B (“PSTGA”), prior to the dissection of the worms. Worms were anaesthetized by incubation at −20 °C for 10 min and decapitated. Then, the animal’s gut was dissected in pre-cooled (4 °C) M-HBSS supplemented with PSTGA, taking care to obtain a highly intact intestine, while avoiding contamination from undesired parts of surrounding tissue. However, due to the tight connection of the intestine, e.g., with chloragogen tissue, a cross contamination of the intestinal tissue with parts of other tissue cannot be completely excluded. The isolated intestine was transferred into fresh, pre-cooled M-HBSS/PSTGA and cleaned of any remaining gut content. The cleaned gut tissue was then transferred into 0.5 mL fresh M-HBSS/PSTGA pre-cooled to 4 °C. The wet weight was determined (ranging between 23 and 162 mg per worm) and the tissue stored on ice until cell isolation.

### Isolation of primary intestinal cells from *Eisenia fetida*

To facilitate the isolation, the gut tissue was digested using collagenase II (90 min, 37 °C in 500 µL M-HBSS/PSTGA and 10 µg/mL collagenase II, under continuous agitation (500 rpm) in an Eppendorf Thermomixer F1.5). After 90 min, the liquid suspension containing the released cells was transferred into a fresh 1.5 mL reaction tube and centrifuged at 200 × *g* for 5 min. The supernatant was removed, the cells were resuspended in 1 mL M-HBSS/PSTGA and filtered through a 20 µm cell strainer. The cells were again pelleted by centrifugation (200 × *g* for 5 min) and resuspended in 100 µL M-HBSS/PSTGA to inoculate the wells of the cell culture plates.

### Cultivation of primary intestinal cells from *Eisenia fetida*

Unless otherwise indicated, freshly isolated cells were cultivated in a medium formulation consisting of 60% (v/v) L-15 medium, 20% (v/v) cell culture grade water, 10% (v/v) FCS, and 10% (v/v) worm filtrate (WF), supplemented with 4 mM L-glutamine, 25 mM HEPES, and PSTGA. The osmolality of the culture media was measured using an Osmomat 030 (Gonotec GmbH, Berlin, Germany) according to the manufacturer’s instructions (indicated reproducibility: <±0.5%). Two standards (0 and 850 mOsmol/kg) were used for calibration. Worm filtrate was sterilized directly before use by 2 × filtration through a sterile filter (0.2 µm cellulose acetate). Cells were seeded at a density of 0.3 to 0.4 × 10^6^ cells/well for 24 well plates and of 0.1 to 0.15 × 10^6^ cells/well for 48 well plates. To reduce evaporation during the cultivation, wells on the edge of the plate were filled with sterile ultrapure water. The plate was incubated in the dark in an airtight box containing a reservoir of sterile ultrapure water at room temperature (20 to 22 °C) for L-15 medium or at 37 °C in a humidified atmosphere containing 5% CO_2_ for Schneider’s medium formulations. To routinely observe cell growth and morphology, an inverse microscope was used (Primovert, Carl Zeiss Microscopy GmbH, Jena, Germany). Images were recorded using an Axiocam 105 color camera and the ZEN 3.0 (blue edition) software. Cell number, size and viability were determined using the automated cell counter LUNA-FL™ Dual Fluorescence Cell Counter (Logos Biosystems, Gyeonggi-do, South Korea). Acridine orange and propidium iodide fluorescence staining was performed according to the manufacturer´s instructions.

### Coating of cell culture plates

To promote cell adherence, various coatings for cell culture plates were tested. The coating procedure was performed under sterile conditions in a biosafety cabinet for 24 well cell culture plates (surface area 1.9 cm^2^ per well); all wells were rinsed with sterile ultrapure water before coating to reduce surface tension. The detailed coating strategy is given in Table S2. After addition of the respective coating solution to the well, the plate was swiveled to distribute the liquid evenly over the entire surface and the plate was incubated for the indicated time at the indicated temperature. Afterwards, the remaining solution was aspirated and—in case of the Poly-L-lysine coating—the well was rinsed four times with sterile ultrapure water. All wells were allowed to dry for 2 h under sterile conditions with half-open lid before introducing medium and cells. Collagen coated wells were rinsed twice with sterile DPBS prior to use.

### MTT-assay for metabolic activity of the primary intestinal cells

0.1 to 0.15 × 10^6^ cells/well in 500 µL culture medium were seeded immediately after isolation in 48 well plates and cultivated for 48 h or 96 h as indicated. 50 µL of a freshly prepared, sterile filtrated solution of 10 mg/mL MTT reagent in M-HBSS buffer were added 24 h before measurement to each well (final MTT concentration: 1 mg/mL) and the cells were further incubated. 24 h after MTT addition, the cells were harvested in reaction tubes and centrifuged at 400 × *g* for 5 min. The supernatant was discarded and the cells containing the formed formazan crystals were resuspended in 250 µL isopropanol. The suspension was split evenly into two wells of a 96 well plate. After 5 min agitating (600 rpm, MS2 Minishaker, IKA, Staufen im Breisgau, Germany), the absorbance at 570 nm (reference wavelength 650 nm) was measured using a TECAN GENios Pro plate reader (Tecan Austria GmbH, Gröding, Austria). The MTT assay was also used to determine the effects of the Ag nanoparticles (1, 3, and 6 µg/mL), the Cu^2+^ (40 and 400 µg/mL) and the Cd^2+^ (80 and 800 µg/mL) ions, as well as the MP particles (2.5 µg MPP and 250 µg per 0.1 × 10^6^ cells) on the isolated intestinal cells. Detailed information of Ag nanoparticles and MP particle concentrations and numbers are given in Tables S3, S4. An additional citrate control (final concentration of 0.6 mM sodium citrate in culture medium) was used to determine effects of the solvent used for Ag nanoparticle suspension and to separate these effects from those of the investigated putative cytotoxins. Ag nanoparticles, metal ions, or MP particles were added immediately after cell seeding. Cells incubated without particles/metal ions were used as a negative control (normalizing condition).

### Microplastic particle uptake studies

For MP particle uptake studies, freshly isolated cells were cultivated for 48 h in the presence of 2.5 µg fluorescent MP particles per 0.1 × 10^6^ cells. Uptake was verified by confocal laser scanning microscopy ((TCS SP8, 63x oil objective, laser: 408 nm, 488 nm, and 552 nm, Leica Microsystems, Wetzlar, Germany). Cells were fixed with 3.7% (v/v) paraformaldehyde in DPBS at 37 °C for 10 min. Afterwards, the cells were centrifuged (180 × *g* for 5 min) and washed once with DPBS. Next, the cells were permeabilized with 0.1% (v/v) Triton X-100 at room temperature for 15 min. After a further wash with DPBS, the cells were stained for 1 h at room temperature with 100 nM rhodamine-phalloidin (Phalloidin-Tetramethyl rhodamine B isothiocyanate, supplier Sigma Aldrich) for actin filament staining and 100 nM DAPI (4′,6-diamidino-2-phenylindole, supplier Sigma Aldrich) for staining of the nuclei. After the staining procedure, another washing step was performed and the cells were seeded in Ibidi slides (Gräfelfing, Germany) for microscopy. The samples were analyzed by confocal laser scanning microscopy; Z-stacks were taken with a step size of 0.33 µm.

### Measurement of ζ-potential and particle size by dynamic light scattering (DLS)

ζ-potential measurements of the particles’ surface charges were performed using the LiteSizer 500 and Omega cuvettes (both Anton Paar Germany GmbH, Ostfildern-Scharnhausen, Germany). 2.5 µL of the MP particle suspensions or 70 µL of the Ag nanoparticle suspension were diluted in 1 mL of a 1 mM aqueous KCl solution (pH 6.0) and measured immediately. In addition, 2.5 µL of the MP particle suspensions or 70 µL of the Ag nanoparticle suspension were incubated in 1 mL of the final culture medium overnight at room temperature. Thereafter, the particles were collected by centrifugation (17,000 × *g*, 40 min) and resuspended in 1 mL of a 1 mM KCl solution for measurement. Three measurements with at least 100 runs each were performed at 21 °C with an adjusted voltage of 200 V. The ζ-potential was calculated using the Helmholtz-Smoluchowski equation (von Smoluchowski 1906). For dynamic light scattering (DLS), 2.5 µL of the MP particle suspensions or 70 µL of the Ag nanoparticle suspension were diluted in 1 mL of a 1 mM aqueous KCl solution (pH 6.0). The measurements with at least 10 runs each at 21 °C in the backscatter mode (angle 175°) were performed using the same device as for the ζ-potential measurements.

### Statistical analyses

All statistical analyses were conducted using the statistical platform R version 4.0.3. (R Core Team 2020). To test the influence of different buffers on viability and cell yield, we used a Kruskal-Wallis test in addition with the *dunn.test* package (Dinno 2017) as a post-hoc comparison. Using a linear model (LM) with the Tukey post-hoc comparison from the *multcomp* package (Hothorn et al. 2008), we tested the impact of different combinations and concentrations of FCS and WF on the metabolic activity of the earthworm intestinal cells. The same procedure was used to check the influence of MP particle, Ag particle, and metal ion exposure on the cells’ metabolic activity. In all whisker boxplots the 25 and 75% quartile are presented with the whiskers representing the maximal and minimal values. Outliers are defined as 1.5 times the value of the 25 and 75% quartile threshold and are represented as points outside the boxplot. Median is indicated as a black line and mean value as a white circle.

## Results and discussion

### Isolation of primary intestinal cells of *Eisenia fetida*

**Fig. 1:**
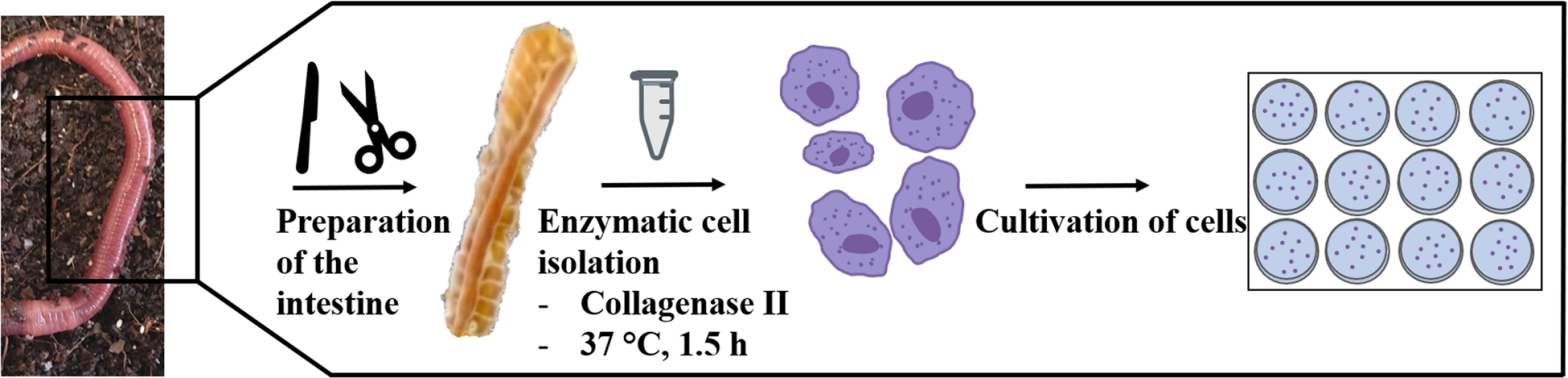
Experimental workflow for the isolation of primary intestinal cells from the earthworm *E. fetida*

In order to obtain a maximum number of vital cells from the intestinal tissue of *E. fetida*, a gentle yet efficient release procedure was established (Fig. 1). Based on previous experience with the release of primary cells from tissue, an enzyme-supported tissue disaggregation step was implemented in the protocol using collagenase II. Collagenase can effectively break down peptide bonds present in collagen, which is the main structural component in the extracellular matrix (ECM) (Rahman 2019; Ricard-Blum 2011).

Composition and in particular osmolality of the buffer receiving the released cells are of major importance for final cell yield and vitality. Here, two buffers were compared for the initial preparation of the intestinal tract as well as the enzymatic treatment. Both buffers, namely M-HBSS and LBSS, had previously been used to collect coelomocytes from *E. fetida* and other earthworm species (Diogène et al. 1997; Engelmann et al. 2004; Eyambe et al. 1991; Irizar et al. 2014; Stein and Cooper 1981). With 210 mOsmol/kg M-HBSS has a slightly higher osmolality than LBSS (171 mOsmol/kg), but both buffers should be in an acceptable range. Further, M-HBSS contains 5.5 mM glucose as potential C-source and 10 mM HEPES as additional buffering agent, whereas LBSS contains no C-source and only very low concentrations (0.4 mM) of phosphate as possible buffering agent (Table S1).

**Fig. 2:**
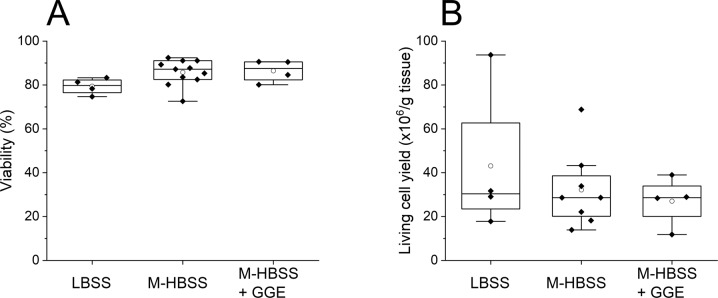
Comparison of LBSS and M-HBSS, as well as M-HBSS, supplemented with 50.4 mM GGE for isolation of primary intestinal cells from *E. fetida*. **A** Viability of the isolated intestinal cells, **B** Living cell yield. *n* ≥ 4

No statistically relevant difference in regard to cell viability could be found between the two buffer systems (Kruskal-Wallis-Test: χ^2^ = 4.42, p-value = 0.11) (Fig. 2A). The isolated cells had a cell size between 5.9 and 20.7 µm (median 11.9 µm, mean 12.6 ± 3.4 µm). The viability was >70% in all cases (n ≥ 4), which we considered sufficient for subsequent cultivations. However, there was a trend towards higher viabilities for cells isolated in M-HBSS (median 87.2% for M-HBSS (n = 11) vs. 79.8% for LBSS (n = 4)). The presence of glucose as a possible carbon source for the isolated cells in case of M-HBSS was most likely responsible for this small but noticeable effect. It is also possible that the higher buffering capacity of M-HBSS helped to stabilize the cells. The living cell yields (Fig. 2B) varied strongly between individual experiments, ranging from 11.8 × 10^6^ to 93.7 × 10^6^ living cells per gram of tissue. The buffer, either M-HBSS or LBSS, again had no significant influence on the average cell yield (Kruskal-Wallis-Test: χ^2^ = 0.68, p-value = 0.71), but the deviations were much more pronounced in case of LBSS. The more easily exhausted buffer capacity of the LBSS buffer may well have contributed to the low reproducibility of the protocol. Therefore, M-HBSS was used in further experiments.

Next, the impact of adding a mucolytic agent during the enzymatic digestion was tested, namely Guaiacol Glyceryl Ether (GGE). GGE is often used for coelomocyte isolation (Diogène et al. 1997; Engelmann et al. 2004; Eyambe et al. 1991) and dissolves in particular mucus-like tissue. When M-HBSS in the absence and presence of GGE (50.4 mM as proposed previously (Diogène et al. 1997)) was used in independent experiments (n ≥ 4), the results showed no significant influence of GGE on the isolation process, as neither viability (Dunn post-hoc comparison: p-value = 0.48) nor living cell yield (Dunn post-hoc comparison: p-value = 0.35) was affected (Fig. 2). Therefore, GGE was not used in subsequent experiments.

### Cultivation of isolated primary intestinal cells from *E. fetida*

For the cultivation of the isolated primary cells, a suitable (basal) culture medium in terms of nutrients, osmolality, buffer system and capacity, as well as pH had to be identified. Moreover, in preliminary cultivation experiments with isolated primary cells, microbial contaminations of the cell culture were observed. This is not surprising, given that earthworms are known for their complex intestinal microbiome (Pass et al. 2015). As earthworms are soil feeders, a high number of bacteria and fungi must be expected in the intestinal tract. Washing steps during the isolation were not sufficient to deplete the microbial burden; presumably microbes were embedded and protected in a mucus layer. Better results were obtained after the addition of a complex mixture of antibiotics (penicillin, streptomycin, tetracycline, gentamycin) together with the antimycotic amphotericin B to the medium (hereafter referred to as PSTGA). PSTGA was supplemented during cell isolation and cultivation and securely prevented microbial contamination for at least 144 h.

Our attempts to identify a suitable basal medium were based on published media formulations (Battaglia and Davoli 1997; Bilej et al. 1990; Engelmann et al. 2004; Gong et al. 2014; Irizar et al. 2014; Roch et al. 1975; Toupin et al. 1977), mostly L-15 and SDM (standard compositions are shown in Table S5), which are summarized in Table 1. Cultivation experiments with L-15 medium were performed at room temperature without additional CO_2_, whereas in case of the SDM based formulations 37 °C and an atmosphere containing 5% CO_2_ were used, as described in literature (Gong et al. 2014; Irizar et al. 2014).

**Table 1:** Media^a^ investigated in the cultivation experiments

	Basal medium (% v/v)	FCS^b^ (% v/v)	Cell culture grade water (% v/v)	Galactose (g/L)	Lactalbumin hydrolysate (g/L)	Osmolality^c^ (mOsmol/kg)
L-15-10	90	10	–	–	–	339 ± 4
L-15-10-GLA	90	10	–	1.3	4.5	345 ± 3
SDM^d^-13	87	13	–	–	–	326 ± 9
SDM-13-GLA	87	13	–		4.5	344 ± 2
Hansen S-301	22	13	65	1.3	4.5	157 ± 5

^a^All media were supplemented with PSTGA. When indicated, media were in addition supplemented with galactose and lactalbumin hydrolysate (GLA) using a concentrated stock solution (200 g/L). L-15 media were supplemented with 4 mM L-glutamine

^b^FCS: Fetal calf serum

^c^Osmolality is shown as mean ± SD (*n* = 5)

^d^SDM: Schneider´s Drosophila Medium

Cell number and viability were analyzed over 144 h of cultivation (data not shown). While no increase in cell number was observed in any of the media formulations over the cultivation time, cell viability remained >90%, showing that the cells could be kept alive for at least 144 h. As no medium was clearly superior to the others, cultivation in L-15 medium at room temperature was chosen as the simplest approach for further experiments. L-15 medium allows cell cultivation without the sodium carbonate/carbon dioxide buffering system as it utilizes free base amino acids (L-arginine, L-histidine, L-cysteine) as buffering agents (Leibovitz 1963). Cultivation at room temperature accommodates *E. fetida* primary cells, since the natural habitat of *E. fetida* is the soil, and laboratory cultures are commonly kept at temperatures between 15 and 20 °C (Miles 1963; Presley et al. 1996; Tripathi and Bhardwaj 2004). For coelomocytes from the congeneric *E. hortensis*, an increase in temperature above 25 °C is known to significantly increase cell death rates (Fuller-Espie et al. 2015). Strikingly, this was not observed here for the intestinal cells, which survived well when cultivated in SDM based media at 37 °C.

The addition of HEPES did not influence cell number or viability (data not shown), but had a beneficial effect on the reproducibility of the experiments, presumably due to the higher buffering capacity. Therefore, the medium was supplemented with HEPES in the subsequent experiments.

Although cell numbers varied slightly during cultivation, significant proliferation was never observed, including the SDM based preparations, which had previously been proposed to support proliferation of intestinal cells from another earthworm species (Gong et al. 2014). Proliferation is not necessary for ecotoxicology experiments, but may be useful in other types of research. The observed lack of proliferation could be explained by a lack of specific growth factors for *E. fetida* cells in the basal culture medium. For primary cells, specific growth factors are typically supplied via blood serum or cell homogenate. Here, an investigative three-step adaption into that direction was performed comprising (1) a supplement screening involving worm filtrate (WF), but also fetal calf serum (FCS) as a standard media additive in mammalian cell culture, (2) an adaption of the osmolality, and finally 3) a verification of the appropriate conditions by measuring the metabolic activity.

**Fig. 3:**
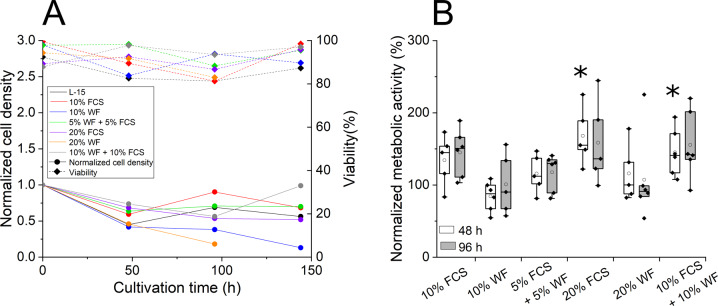
Cultivation of primary intestinal cells of *E. fetida* in media formulations containing the indicated amounts of FCS and/or WF. **A** Representative cultivations with normalized cell density to the seeding cell density and viability for cultivation in the indicated media over time. **B** Metabolic activity of primary intestinal cells after 48 and 96 h of cultivation in the indicated media. Shown is the metabolic activity normalized to L-15-60% without FCS and/or WF supplementation. *n* ≥ 5. Statistically significant differences to the negative control are indicated by *

First, we recorded cell number and viability at different concentrations and combinations of FCS and WF in cultivation experiments (Fig. 3A). L-15 medium without additive yielded a slight reduction of cellular viability over time, albeit never dropping below 80%. Both FCS or WF appeared to slightly improve cell viability. Since there was no clear difference between WF and FCS as additive, we chose a mixture of 10% FCS and 10% WF as starting composition for the osmolality adaption, evaluating different dilutions of L-15 medium for cultivation, corresponding to final osmolalities between 308 and 381 mOsmol/kg (Table S6). In the subsequent cultivation experiments, there was no clear influence of the medium dilutions on cell number or viability (data not shown). For the common earthworm *Aporrectodea caliginosa*, a broad range of body fluid osmolality from 175 to 684 mOsmol/kg was measured for different dehydration states suggesting a high tolerance of that species against a broad range of osmolalities (Bayley et al. 2010). Therefore, L-15-60% (10% v/v FCS and WF, 60% v/v L-15, 20% v/v cell culture grade water) was chosen for further experiments, as its osmolality of approximately 310 mOsmol/kg was considered to be closest to characteristic osmolalities of terrestrial animals (Stankiewicz and Plytycz 1998).

Cellular vitality, i.e., metabolic activity, is an equally important indicator for cell cultivation and toxicity testing. Using L-15-60% as basis, the impact of the FCS/WF supplement on cellular vitality was investigated using the MTT assay as analytical tool (Fig. 3B). Cells cultivated in 10% FCS + 10% WF or 20% FCS reached the highest mitochondrial metabolic activity, i.e., 150% compared to that in L-15-60% without FCS/WF supplementation. The difference was statistically significant (LM_Treatment_ F_5_ = 4.706, p-value < 0.004, Tukey post-hoc comparison: p-value_10% FCS + 10% WF_ = 0.027, p-value_20% FCS_ = 0.002). L-15-60%, supplemented with 10% FCS, 10% WF, as well as 4 mM L-glutamine, 25 mM HEPES, and PSTGA was therefore chosen as standard culture medium for *E. fetida* cells.

**Fig. 4:**
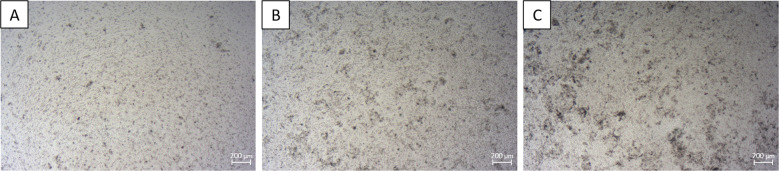
Representative microscopical images of primary intestinal cells seeded at different cell densities after 48 h of cultivation in 24 well plates. **A** Cell seeding density: 0.053 × 10^6^ cells/cm^2^. **B** Cell seeding density: 0.210 × 10^6^ cells/cm^2^. **C** Cell seeding density: 0.421 × 10^6^ cells/cm^2^. Scale bar = 200 µm

Cell seeding density is a critical factor for primary cell cultivation, as a sufficient number of cells is needed for cell-cell interactions as well as for the production of autocrine growth factors. However, in the case of primary cells, proliferation often stops once confluency is reached during cultivation. Therefore, the effect of seeding cell densities was analyzed microscopically for seeding cell densities between 0.053 × 10^6^ cells/cm^2^ and 0.421 × 10^6^ cells/cm^2^ in 24 well plates with 1 mL culture medium (Fig. 4).

In these experiments, cell seeding densities between 0.158 × 10^6^ cells/cm^2^ and 0.210 × 10^6^ cells/cm^2^ were optimal. Lower seeding densities led to large, uncovered areas, whereas higher cell densities resulted in an increased number of floating cell aggregates after 48 h. This seeding density is in accordance with previously published results using *P. aspergillum* primary epithelial cells (Gong et al. 2014).

The occurrence of floating cellular aggregates indicated that the cells failed to properly adhere to the cell culture plate. Adherence is an important factor for growth of intestinal cells considering that these cells are derived from epithelial tissue. To promote adherence of the isolated cells to the culture plate, different coating strategies were evaluated based on standard cell culture coating materials, in particular poly-L-lysine, gelatin (porcine), collagen type I (human) and collagen type II (bovine) (Davidenko et al. 2016; Harnett et al. 2007; Liberio et al. 2014). None of the treatments improved cell adhesion. We currently assume that the lack of suitable adhesion factors is a major contribution to the lack of proliferation observed for the intestinal cells.

### Ecotoxicity testing using primary intestinal cells of *E. fetida*

**Fig. 5:**
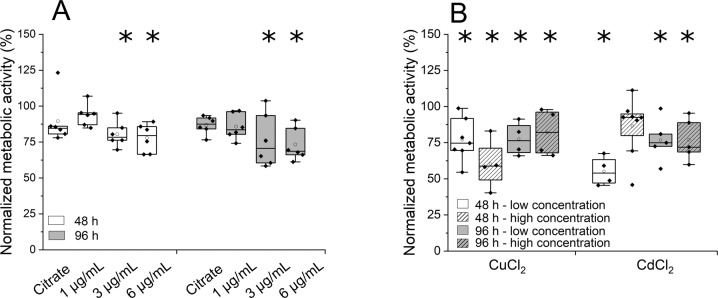
Influence of (**A**) Ag nanoparticles and (**B**) metal ions on the metabolic activity of primary intestinal cells from *E. fetida* analyzed using the MTT assay after 48 and 96 h of incubation. Shown is the metabolic activity of cells normalized to a negative control (cells incubated without particles or metal ions). *n* ≥ 3. Statistically significant differences to the negative control) are indicated by *. CuCl_2_, low concentration: 40 µg/mL, high concentration: 400 µg/mL CdCl_2,_ low concentration: 80 µg/mL, high concentration: 800 µg/mL

Even in the absence of proliferation, highly viable, metabolically active primary cells present an excellent basis for a study of acute toxic effects of common ecotoxins on the cellular level. For a demonstration, we examined the influence of known environmental pollutants, namely Ag nanoparticles and metal ions (Cu^2+^, Cd^2+^) on the metabolic activity of the isolated cells (Fig. 5).

For the intestinal cells, exposure to Ag nanoparticles led to a slight, but significant decrease in metabolic activity (LM_Treatment_ F_9_ = 4897; p < 0.001) in a concentration-dependent manner compared to the negative control (cells in culture medium) (Tukey post-hoc comparison: p-value_48 h_ = 0.130, p-value_96 h_ = 0.084) (Fig. 5A). Cells exposed to the citrate buffer used to suspend the Ag nanoparticles (“citrate control”, amount corresponding to 6 µg/mL Ag nanoparticles) also showed a reduced metabolic activity, but the effect was clearly enhanced in presence of 3 or 6 µg/mL Ag nanoparticles. On the organismic level, Ag nanoparticles show only slight to negligible effects on traditional ecotoxicological endpoint markers like growth, mortality and reproduction of *E. fetida* (Kwak et al. 2014; Shoults-Wilson et al. 2011). Kwak et al. (2014) demonstrated the importance of the nanoparticular material, since Ag nanoparticles showed a slight effect on *E. andrei* while the exposure to pure Ag ions showed no effect at all. On the other hand, isolated coelomocytes of *E. fetida* cultivated in RPMI-1640 medium showed an LC_50_ value of 6 µg/mL (Garcia-Velasco et al. 2019). Coelomocytes cultivated in L-15 medium, on the other hand, showed a much higher resistance to Ag nanoparticles (LC_50_ > 100 µg/mL) (Garcia-Velasco et al. 2019). Differences between the studies may stem from differences in particle size as well as different cell types used and therefore non-comparable cellular reactions. In the end only additional studies on the cellular level can elucidate the mechanistic basis for the observed toxic effects.

In contrast to particles, whose size and surface coverage are known to have a significant effect on toxicity, metal ions are considered to be more standardizable toxins. The response of the cells to copper and cadmium ions are summarized in Fig. 5B. The results show a significant decrease in metabolic activity for all tested concentrations (LM_Treatment_ F_8_ = 5.027, p < 0.001), except 800 µg/mL CdCl_2_ after 48 h exposure. After 96 h the cells seemed to recover to some extent, but the metabolic activity was still low compared to the controls. A more pronounced effect had been expected in particular for the respective higher metal ion concentrations, i.e., 400 µg/mL CuCl_2_ and 800 µg/mL CdCl_2_, since these are already in the range of the median lethal concentration for *E. fetida* in soil, namely 500–700 mg/kg soil for copper and 600–1800 mg/kg soil for cadmium (Bernard et al. 2015; Neuhauser et al. 1985). Interestingly, coelomocytes isolated from *E. fetida* showed a similar response at least to changing cadmium doses, where the viability decreased in the presence of 100 µg/mL followed by an increase in viability at concentrations of 500 µg/mL (Irizar et al. 2015). Irizar et al. (2015) assumed that different stress mechanism exist for *E.fetida*, which might also play a role in the recovery of the cells after a longer incubation with Cd. However, no specific mechanisms are known yet. The discrepancy between organismic effects and the reactions described here suggest different toxicological mechanisms on different levels of biological complexity. Our work provides a basis to analyze these mechanisms on a cellular level for intestinal cells.

### Effects of MP particles on primary intestinal cells isolated from *E. fetida*

Finally, the influence of MP particles was investigated on the cells as an example of a new and increasingly important environmental pollutant with particular relevance for unspecific soil feeders such as *E. fetida*. PS microparticles were chosen as representatives of non-biodegradable commodity plastics, whereas PLA microparticles were chosen as example for a biodegradable polymer. The possibility of a cellular uptake of the particles was also studied, using particle sizes between 0.2 and 3 µm.

Surface properties and in particular a biomolecular corona on the particle’s surface have recently been suggested as decisive for cell particle interaction and uptake (Ramsperger et al. 2020). Therefore, once the MP particles are added to the cells in the protein-rich culture medium the formation of a protein corona is likely. ζ-potentials and size distribution measured of MP particles incubated at various conditions are summarized in Table 2.

**Table 2:** ζ-potential and size distribution measured for the MP particles used in this study

Nominal particle size (µm)	ζ-potential (mV)		Measured particle size (µm)	
	KCl	Culture medium	KCl	Culture medium
PS				
0.2	−47.4 ± 0.3	−25.3 ± 0.0	0.2 ± 0.006	0.2 ± 0.008
0.5	−52.8 ± 0.2	−27.6 ± 0.0	0.5 ± 0.008	0.6 ± 0.04
2	−76.7 ± 0.3	−28.8 ± 0.0	1.8 ± 0.03	1.5 ± 0.04
3	−78.9 ± 0.3	−29.3 ± 0.2	3.1 ± 0.08	3.3 ± 0.1
PLA				
0.5	−1.1 ± 0.0	−1.3 ± 0.4	0.6 ± 0.02	1.1 ± 0.1
2	−3.9 ± 0.3	−11.2 ± 1.1	1.5 ± 0.2	2.4 ± 1.2

Incubation in culture medium led to a reduction of the ζ-potential. Even particles with significantly different ζ-potential before incubation showed similar ones after incubation in culture medium independent of the particle diameter. This indicates the development of a similar protein corona on the surface of all investigated PS particles. Pristine PLA particles, on the other hand, initially showed a small negative ζ-potential, which was slightly increased in case of the 2 µm particles after incubation. As expected, the PLA particles were colloidally instable due to the low ζ-potential, and thereby the size of the PLA particles nearly doubled after incubation in the culture medium and the size distribution became wider.

**Fig. 6:**
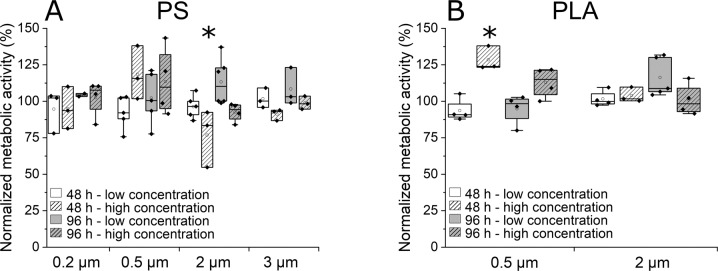
Influence of MP particles (MPP) on the metabolic activity of primary intestinal cells from *E. fetida* after 48 and 96 h incubation time. **A** Influence of PS particles on cells incubated with 2.5 µg MPP/0.1 × 10^6^ cells and 250 µg MPP/0.1 × 10^6^ cells. **B** Influence of PLA particles on cells incubated with 2.5 µg MPP/0.1 × 10^6^ cells and 250 µg MPP/0.1 × 10^6^ cells. Shown is the metabolic activity of cells normalized to a negative control (cells incubated without particles) analyzed using the MTT assay. *n* ≥ 3. Statistically significant differences to the negative control are indicated by *

Within the 24 exposure experiments (Fig. 6), only cells incubated at high concentrations of 2 µm PS particles showed a significantly reduced metabolic activity after 48 h of incubation (LM_Treatment_ F_24_ = 2.291, p = 0.004, Tukey post-hoc comparison: p-value_2 µm PS_ = 0.025). In the presence of 0.5 µm PLA at the high concentration after 48 h the cells even showed a significantly higher metabolic activity (LM_Treatment_ F_24_ = 2.291, p = 0.004, Tukey post-hoc comparison: p-value_0.5 µm PLA_ = 0.006). This finding seems counterintuitive, however correlations between dose and response may not necessarily be linear as already shown for *E. fetida* on the organismic level (Chen et al. 2020; Jiang et al. 2020), as well as for coelomoycetes (Irizar et al. 2015).

**Fig. 7:**
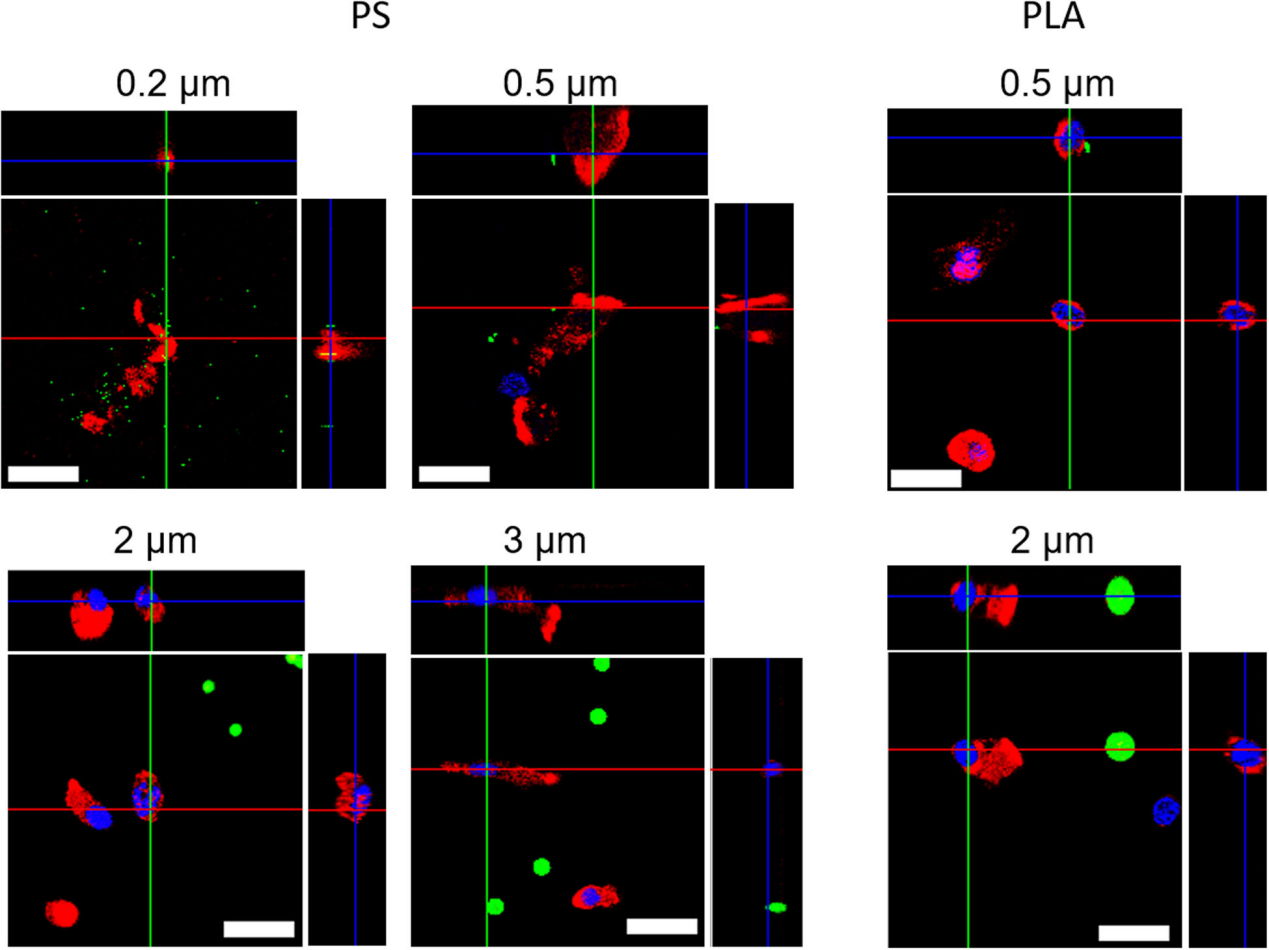
Confocal laser scanning microscopy images of primary intestinal *E. fetida* cells incubated in presence of 2.5 µg MPP/0.1 × 10^6^ cells for 48 h. The actin filaments were stained with rhodamine-phalloidin (red), nuclei were stained with DAPI (blue) and the FITC-fluorescent MP particles showed a green fluorescence. Scale bar = 20 µm

Finally, confocal microscopy showed no apparent uptake of fluorescent MP particles of any type and size by the cells. Particles ≤0.5 µm showed some tendency of attachment to the cellular membranes, however, no signs of uptake or attachment were seen for larger particles (Fig. 7). This might also explain the observed low effect of the particles on the metabolic activity of the cells. Moreover, these findings are in line with recent results for murine epithelial cell lines, where also no uptake of particles >0.2 µm was observed (Rudolph et al. 2021). Particles with a size < 1 µm already constitute nano/submicromaterials. The preference for their uptake by cells from various species stresses the need to extent the research on microplastic in the environment to that of nanoplastic (NP). In particular, it was show for a fish cell line that the synergistic effect of nanoparticles with environmental relevant metals like arsenic and methylmercury increases the cytotoxicity compared to the respective single effects (González-Fernández et al. 2021). Since the smallest investigated particles (0.2 µm) seem to attach to cells, possible secondary or cumulative effects cannot be excluded. Organismic effects, like tissue damage or the inflammation of the gut tissue as shown previously (Jiang et al. 2020) might derive from particles which are not taken up by cells but persist in extracellular spaces in the tissue.

## Conclusion

Establishing primary cells of model organisms for ecotoxicological studies is challenging, yet paves the way to mechanistic studies of the toxic effects on the cellular level. Here, we establish a method for the cultivation of primary intestinal cells of the earthworm *E. fetida*. Cells were kept viable and metabolically active for at least 144 h. This is sufficient time to study cellular responses in detail, including in future also changes on the transcriptomic and metabolomic level. Utility for ecotoxicological tests on the cellular level was shown using known toxic agents. In contrast to the cytotoxic effects induced by these agents, MP particles neither induced any negative effects on the metabolic activity nor could active uptake of the particles be observed by the primary intestinal cells. In consequence, the established isolation method for intestinal primary cells from *E. fetida* allows more detailed studies on the cellular level to enhance our understanding how toxic effects of environmental pollutants are mediated on the organismic level.

## Supplementary information

Supplementary Information
