# Supplementary material for: In vitro cultivation of primary intestinal cells from Eisenia fetida as basis for ecotoxicological studies
Source: Ecotoxicology. 2021 Nov 17;31(2):221–33. doi: 10.1007/s10646-021-02495-2 (PMC8901508; doi:10.1007/s10646-021-02495-2)
Supplement: Supplementary file 1 — Supplementary Information [file 10646_2021_2495_MOESM1_ESM.docx]

**Supporting information**

**Ex vivo cultivation of primary intestinal cells from *Eisenia fetida* as basis for ecotoxicological studies**

Ecotoxicology

Simon A. B. Riedl^a1^, Matthias Völkl^a1^, Anja Holzinger^b,^ Julia Jasinski^c^ ,Valérie Jérôme^a^, Thomas Scheibel^c^, Heike Feldhaar^b^, Ruth Freitag^a*^

^1^: Both authors contributed equally

^a^Process Biotechnology, ^b^Animal Ecology I, Bayreuth Center of Ecology and Environmental Research (BayCEER), ^c^Biomaterials, University of Bayreuth, 95440 Bayreuth, Germany

*corresponding author, e-mail address: ruth.freitag@uni-bayreuth.de, postal address: Process Biotechnology, University of Bayreuth, 95440 Bayreuth, Germany

**Table S1** Detailed composition and characteristics of M-HBSS and LBSS. M-HBSS was described by Diogène et al. (1997) and LBSS by Stein and Cooper (1981)

|  | M-HBSS | LBSS |
| --- | --- | --- |
| pH | 7.25 | 7.3 |
| Osmolality (mOsmol/kg) | 210 | 171 |
| KCl (mM) | 5.3 | 4.8 |
| KH_2_PO_4_ (mM) | 0.396 | 0.4 |
| NaHCO_3_ (mM) | 4.09 | 4.2 |
| Na_2_HPO_4_ (mM) | 0.298 | - |
| Glucose (mM) | 5.5 | - |
| HEPES (mM) | 10 | - |
| NaCl (mM) | 84.9 | 71.5 |
| CaCl_2_ (mM) | - | 3.8 |
| MgSO_4_ (mM) | - | 1.1 |
| Na_2_HPO_4_ (mM) | - | 0.4 |

**Table S2** Recipes used for coating of 24 well tissue culture plates

|  | Poly-L-lysine | Gelatin (porcine) | Collagen type I (human) | Collagen type II (bovine) |
| --- | --- | --- | --- | --- |
| Concentration (µg/mL) | 5000 | 1000 | 20 | 7.6 |
| Solvent | cell culture grade water | cell culture grade water | 10 mM HCl | 500 mM acetic acid |
| Volume added per well (µL) | 76 | 190 | 475 | 500 |
| Coating concentration (µg/cm^2^) | 200 | 100 | 5 | 2 |
| Incubation time (min) | 5 | 120 | overnight | overnight |
| Incubation temperature (°C) | RT^a^ | 37 | RT | RT |

^a^RT: room temperature

**Table S3** Numbers of Ag nanoparticles used in MTT assays to analyze their influence on the metabolism of the isolated primary intestinal cells

| Particles | Concentration (µg/mL) | Number of particles / µg | Number of particles/ 0.1 x 10^6^ cells |
| --- | --- | --- | --- |
| 40 nm AgNP | 1 | 2.83 x 10^9^/ 1 µg | 2.83 x 10^4^ |
| 40 nm AgNP | 3 | 8.48 x 10^9^ / 3 µg | 8.48 x 10^4^ |
| 40 nm AgNP | 6 | 1.70 x 10^10^ / 6 µg | 1.70 x 10^5^ |

**Table S4** Numbers of MP particles used in MTT assays to analyze their influence on the metabolism of the isolated primary intestinal cells

| Particles | Low concentration (2.5 µg MPP/ 0.1 x 10^6^ cells) | | High concentration (250 µg MPP/ 0.1 x 10^6^ cells) | |
| --- | --- | --- | --- | --- |
|  | Number of particles/ 2.5 µg | Number of particles/ 0.1 x 10^6^ cells | Number of particles/ 250 µg | Number of particles/ 0.1 x 10^6^ cells |
| 0.2 µm PS | 5.74 x 10^8^ | 5.74 x 10^3^ | 5.74 x 10^10^ | 5.74 x 10^5^ |
| 0.5 µm PS | 3.67 x 10^7^ | 3.67 x 10^2^ | 3.67 x 10^9^ | 3.67 x 10^4^ |
| 2 µm PS | 5.74 x 10^5^ | 5.74 | 5.74 x 10^7^ | 5.74 x 10^2^ |
| 3 µm PS | 1.70 x 10^5^ | 1.70 | 1.70 x 10^7^ | 1.70 x 10^2^ |
| 0.5 µm PLA | 3.67 x 10^7^ | 3.67 x 10^2^ | 3.67 x 10^9^ | 3.67 x 10^4^ |
| 2 µm PLA | 5.74 x 10^5^ | 5.74 | 5.74 x 10^7^ | 5.74 x 10^2^ |

**Table S5** Compositions of L-15 medium and Schneider´s Drosophila Medium. L-15 medium was described by Leibovitz (1963) and Schneider´s Drosophila Medium by Schneider (1964)

|  | L-15 | Schneider´s Drosophila Medium |
| --- | --- | --- |
| pH | 7.4 - 7.9 | 6.2 - 6.8 |
| Osmolality (mOsmol/kg) | 300 – 340 | 320 -340 |
| Carbon source | 0.9 g/L galactose | 2 g/L glucose |
| L-glutamine (mM) | - | 12 |
| KH_2_PO_4_ (g/L) | 0.06 | 0.45 |
| Na_2_HPO_4_ (g/L) | 0.19 | 0.70 |
| NaHCO_3_ (g/L) | - | 0.40 |

**Table S6** Medium composition^a^ based on different dilutions of basal medium L-15

| Medium composition | L-15 (% v/v) | Cell culture grade water (% v/v) | Osmolality (mOsmol/kg) |
| --- | --- | --- | --- |
| L-15-80% | 80 | 0 | 381 ± 10 |
| L-15-70% | 70 | 10 | 347 ± 3 |
| L-15-60% | 60 | 20 | 308 ± 11 |

^a^All media were supplemented with 10% (v/v) FCS and 10% (v/v) WF, 4 mM L-glutamine, 25 mM HEPES, and PSTGA (100 U/mL penicillin, 100 µg/mL streptomycin, 60 µg/mL tetracycline, 50 µg/mL gentamycin and 2.5 µg/mL amphotericin B). Osmolality is shown as mean ± SD (n = 5).

**References**

Diogène J, Dufour M, Poirier GG, Nadeau D (1997) Extrusion of earthworm coelomocytes: comparison of the cell populations recovered from the species *Lumbricus terrestris*, *Eisenia fetida* and *Octolasion tyrtaeum*. Lab Anim 31:326–336. https://doi.org/10.1258/002367797780596068

Leibovitz A (1963) The growth and maintenance of tissue-cell cultures in free gas exchange with the atmosphere. Am J Hyg 78:173–180. https://doi.org/10.1093/oxfordjournals.aje.a120336

Schneider I (1964) Differentiation of larval drosophila eye-antennal discs in vitro. J Exp Zool 156:91–103. https://doi.org/10.1002/jez.1401560107

Stein E, Cooper EL (1981) The role of opsonins in phagocytosis by coelomocytes of the earthworm, *Lumbricus Terrestris*. Dev Comp Immunol 5:415–425. https://doi.org/10.1016/S0145-305X(81)80054-7
